# Efficacy and safety of acupuncture for post-stroke spasticity: a study protocol for a randomized controlled trial

**DOI:** 10.3389/fneur.2026.1737118

**Published:** 2026-06-04

**Authors:** Zhihao Xiong, Juwei Dong, Yingying Zhu, Jiaxu Liu, Yini Hua, Yue Song, Jinxia Ni, Liangxiao Ma, Jing Bai

**Affiliations:** 1 Dongzhimen Hospital, Beijing University of Chinese Medicine, Beijing, China; 2 School of Acupuncture-Moxibustion and Tuina, Beijing University of Chinese Medicine, Beijing, China; 3 The Key Unit of State Administration of Traditional Chinese Medicine, Evaluation of Characteristic Acupuncture Therapy, Beijing, China

**Keywords:** acupuncture, post-stroke spasticity, randomized controlled trial, sham acupuncture, study protocol

## Abstract

**Background:**

Post-stroke spasticity (PSS) is a common motor complication during stroke recovery, imposing a substantial burden on both individuals and society owing to its high morbidity and disability rates. Current evidence suggests that acupuncture may be an effective intervention for alleviating PSS; however, the efficacy and safety remain uncertain owing to methodological limitations and the generally low quality of the existing evidence. Therefore, this study aims to evaluate the efficacy, feasibility, and safety of acupuncture for PSS and to explore the underlying anti-spasticity mechanisms.

**Methods:**

This study is a prospective, randomized, controlled trial. A total of 180 patients diagnosed with post-stroke spasticity (PSS) will be enrolled and randomly assigned in a 1:1:1 ratio to one of the three groups: acupuncture, sham acupuncture, or the basic treatment group. Participants in the acupuncture and sham acupuncture groups will receive a 4-week intervention, followed by a 4-week follow-up period. In contrast, the basic treatment group will receive only conventional medical management and standardized rehabilitation training without any acupuncture-related procedures. The primary outcome measure will be spasticity severity, assessed using the Modified Ashworth Scale (MAS). The secondary outcomes will include motor function (Fugl-Meyer Assessment, FMA), clinical spasticity (Clinical Spasticity Index, CSI), neurological impairment (National Institutes of Health Stroke Scale, NIHSS), activities of daily living (Modified Barthel Index, MBI), neuromuscular activity (surface electromyography, sEMG), and muscle mechanical properties (ultrasonographic elastography, UE). The FMA, CSI, NIHSS, and MBI will be evaluated at baseline, week 2, week 4 (end of treatment), and the 4-week follow-up visit. The MAS, sEMG, and UE will be assessed at baseline and at the end of the 4-week treatment period.

**Discussion:**

The results of this trial will help to clarify the preliminary efficacy, clinical feasibility, and safety of acupuncture for PSS patients and explore the anti-spastic mechanisms of acupuncture.

**Clinical trial registration:**

https://itmctr.ccebtcm.org.cn/mgt/project/view/1963190034123325440, ITMCTR2025001662.

## 1 Introduction

Stroke is the second leading cause of death from non-communicable diseases (NCDs) and the third leading cause of disability-adjusted life-years (DALYs) globally (1). Stroke-induced motor dysfunction manifests in diverse forms, including paresis/weakness, spasticity, impaired motor control, and abnormal synergies. Among these impairments, spasticity is a prevalent and clinically significant feature during stroke recovery and is considered a hallmark of upper motor neuron syndrome (2). A literature review reveals that the median time to onset of post-stroke spasticity (PSS) is 34 days following the index stroke event (3). In the chronic phase, spasticity affects up to 97% of stroke survivors, with moderate to severe motor impairment (4). Given its high prevalence and chronic nature, PSS substantially increases therapeutic complexity, markedly impairs activities of daily living (ADLs), and imposes a considerable burden on both caregivers and healthcare systems. Therefore, elucidation of the underlying pathogenic mechanisms and development of safe, effective, and evidence-based interventions for PSS represent critical unmet clinical needs.

Originally proposed in 1980, the clinical term “spasticity” was defined by J. W. Lance as “a motor disorder characterized by a velocity-dependent increase in tonic stretch reflex activity (i.e., muscle tone) and exaggerated tendon jerks, resulting from hyperexcitability of the stretch reflex—a key feature of the upper motor neuron syndrome (UMNS)” (5). Owing to the complexity of its pathogenic factors, several hypotheses have been proposed regarding the pathogenesis of PSS. Current evidence indicates that spasticity reflects aberrant neural plasticity arising from disrupted sensorimotor integration; specifically, hyperactive stretch reflexes enhance neuronal excitability, thereby accelerating the progression of spasticity (6). Additional contributors, including passive muscle stretching, local inflammation, and tissue injury, can alter the biomechanical properties of skeletal muscles and tendons. These changes increase the sensitivity of muscle spindles and other proprioceptive receptors, thereby enhancing sensorimotor feedback, increasing motor neuron firing rates, and promoting hypersensitivity responses of muscles and tendons to sensory stimuli, which collectively exacerbate spasticity (7). Furthermore, the increased release of neurotransmitters, such as glutamate (Glu), acetylcholine (ACh), and 5-hydroxytryptamine (5-HT), can prolong involuntary muscle contractions and extend the duration of spasticity (8).

Treatment of PSS focuses on the control of spasticity and improvement of motor function, with an emphasis on early intervention (within 3 months after stroke). Current therapeutic approaches encompass both pharmacological and non-pharmacological modalities. Pharmacological interventions include intramuscular botulinum toxin injections and oral anti-spasmodic agents such as baclofen; non-pharmacological options include acupuncture, exercise therapy, mirror therapy, and extracorporeal shock wave therapy (9). Baclofen, a *γ*-aminobutyric acid (GABA)-b agonist, is the most commonly used oral anti-spasmodic with generally good efficacy, but it has certain adverse effects, including drowsiness, fatigue, hepatotoxicity, and, in some cases, paradoxical muscle weakness (10). Botulinum toxin type A (BoNT-A) intramuscular injection is an effective therapy for relieving spasm-related pain; however, its high cost may limit its wider application (11). Given the limitations of existing anti-spasticity agents, there is a pressing need to investigate new therapeutic strategies that can simultaneously enhance efficacy and improve safety in treating PSS patients.

Research indicates that acupuncture ameliorates motor dysfunction by activating the primary motor cortex (M1) of the ipsilesional hemisphere and promoting brain network remodeling in motor-related areas (12). Acupuncture is currently used to treat PSS. Clinical evidence suggests that acupuncture can alleviate spasticity, enhance motor function, and accelerate rehabilitation and has received a Grade B recommendation for PSS management (13). Experimental studies indicate that acupuncture ameliorates PSS through multiple mechanisms. It modulates the Glu/GABA–glutamine (Gln) cycle via glutamate transporter 1 (GLT-1), thereby rebalancing Glu/GABA levels and mitigating spinal hyperreflexia (14). In addition, it enhances cerebral blood flow in the ischemic penumbra and restores spinal inhibitory control via 5-hydroxytryptamine receptor 2A (5-HT_2A_R)-dependent potassium–chloride cotransporter 2 (KCC2) reactivation, thereby improving limb spasticity (15). However, the majority of the studies are of low methodological quality and lack standardized acupuncture protocols and rigorous study designs. In addition, the potential placebo effects remain incompletely characterized, which may hinder definitive conclusions. Further high-quality research is essential to validate the efficacy and safety of acupuncture for PSS and to elucidate the underlying neuromuscular mechanisms, providing more reliable, evidence-based guidance for personalized prophylaxis and therapy for PSS.

As a solution, we designed a pilot randomized controlled trial (RCT) to assess the preliminary efficacy, feasibility, and safety of acupuncture for PSS and explore the underlying anti-spasticity mechanism of acupuncture.

## 2 Methods and design

### 2.1 Study design

**Figure 1:**
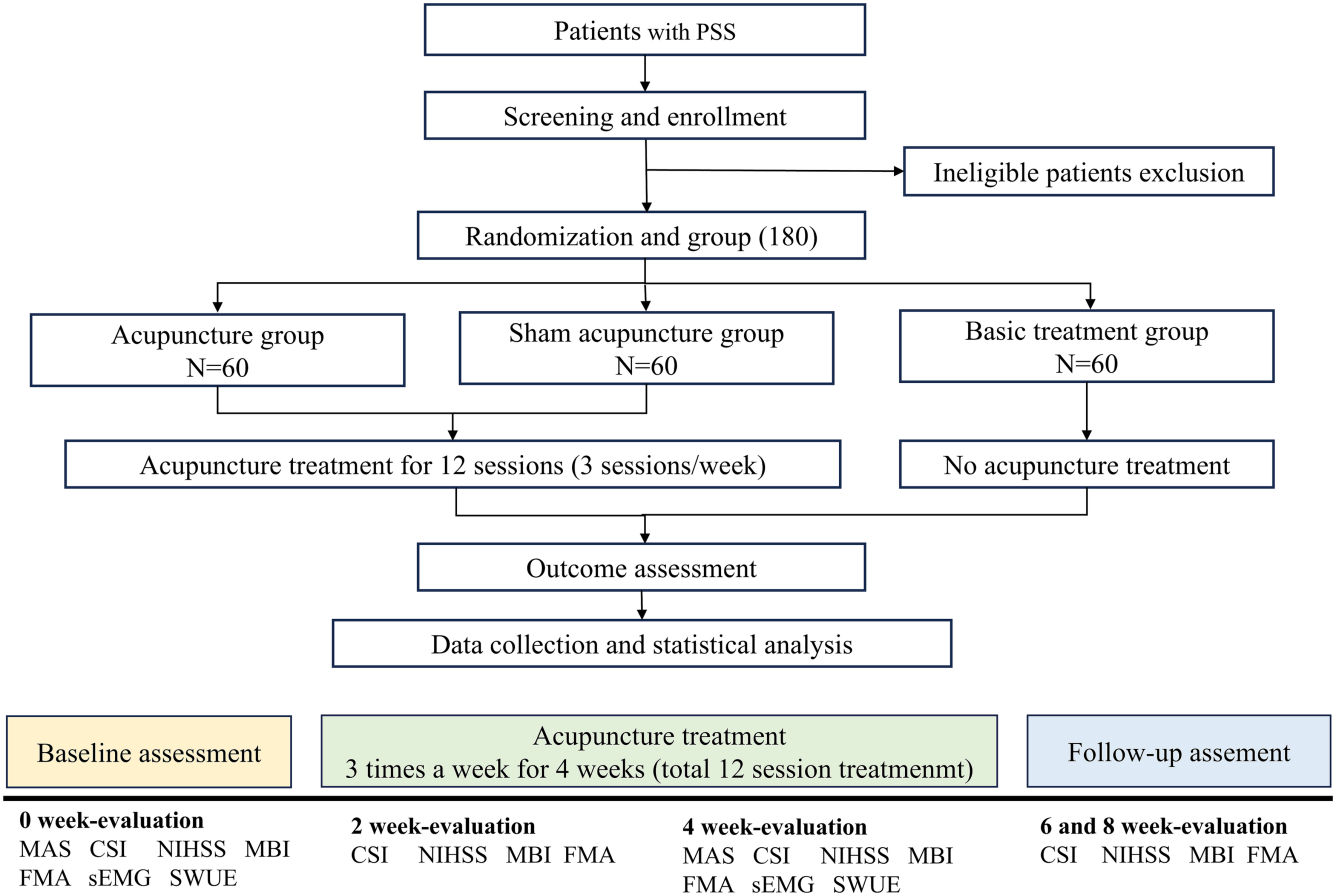
Study flowchart. MAS, Modified Ashworth Scale; CSI, Clinical Spasticity Index; NIHSS, National Institutes of Health Stroke Scale; FMA, Fugl-Meyer Motor Assessment; MBI, Modified Barthel Index; sEMG, Surface Electromyography; SWUE, Shear-wave ultrasound elastography.

This study is a 4-week randomized controlled trial, in which 180 participants will be randomly allocated to three groups in a 1:1:1 ratio. The trial will be conducted from December 2024 to December 2026 at Dongzhimen Hospital, Beijing University of Chinese Medicine. Both the acupuncture and sham acupuncture groups will receive standard conventional therapy and rehabilitation training, along with 12 sessions of acupuncture (administered three times per week) over a 4-week treatment period. The control group—referred to as the basic treatment group—will receive only conventional therapy and rehabilitation training during the 4-week intervention period, without any acupuncture. As compensation, the participants in this group will receive 12 sessions of acupuncture after an 8-week follow-up period. The trial protocol will be based on the Standard Protocol Items: Recommendations for Interventional Trials (SPIRIT) checklist. This study has been approved by the Ethics Committee of Dongzhimen Hospital, Beijing University of Chinese Medicine, and has been prospectively registered with the International Traditional Medicine Clinical Trial Registry (ITMCTR). The study flowchart is presented in Figure 1, and the timeline for participant enrollment, interventions, and outcome assessments is detailed in Table 1.

**Table 1:** Schedule of enrollment, interventions, and assessments.

Time points	Enrollment	Baseline	Treatment period				Follow-up period	
	Week 1	Week 0	Week 1	Week 2	Week 3	Week 4	Week 6	Week 8
Screening and recruitment								
Inclusion and exclusion	x							
Clinical interview	x							
Sign the informed consent	x							
Randomization		x						
Interventions								
Acupuncture group			12 sessions of treatment (3 times a week)					
Sham acupuncture group			12 sessions of treatment (3 times a week)					
Basic treatment group			No acupuncture treatment					
Assessments								
MAS		x		x		x	x	x
FMA		x		x		x	x	x
CSI		x		x		x	x	x
NIHSS		x		x		x	x	x
MBI		x		x		x	x	x
sEMG		x				x		
SWUE		x				x		
Others								
Adverse events			x	x	x	x		
Blinding assessment			x	x	x	x		
Patients’ compliance			x	x	x	x		
Type of stroke		x						
Patients’ complications		x						

MAS, Modified Ashworth scale; FMA, Fugl-Meyer motor function assessment; CSI, comprehensive spasticity scale; NIHSS, National Institutes of Health Stroke Scale; MBI, Modified Barthel Index; sEMG, surface electromyography; SWUE, Shear-wave ultrasound elastography.

### 2.2 Participants

#### 2.2.1 Inclusion criteria

Participants who meet all conditions will be considered for enrollment. The inclusion criteria are as follows: (1) a diagnosis of ischemic or hemorrhagic stroke confirmed by cranial CT/MRI. (2) First-time stroke or recurrent stroke without residual neurological impairment. (3) Duration: 2 weeks to 6 months after stroke onset. (4) Male or female patients aged between 35 and 75 years. (5) Patients met the diagnostic and assessment criteria for PSS: muscle stiffness, shortening, and fixed joint contractures, with grades 1–3 assessed by the Modified Ashworth Scale (MAS) (16). (6) A National Institutes of Health Stroke Scale (NIHSS) score between 4 and 16. (7) Conscious patients (Glasgow Coma Scale score = 15) are able to participate in medical interviews, physical examinations, instructions, and treatment. (8) Patients who agreed to participate in the trial and provided written informed consent.

#### 2.2.2 Exclusion criteria

Patients with any one of the following criteria will be excluded from the trial: (1) patients with severe cardiovascular diseases, such as decompensated heart failure (New York Heart Association [NYHA] class III-IV), myocardial infarction (within the last 6 months), or unstable angina pectoris. (2) Immunological/hematological diseases: active autoimmune disease requiring long-term immunosuppression (e.g., >10 mg/day prednisone-equivalent dose) or uncontrolled coagulopathy (e.g., International Normalized Ratio [INR] > 3.0, not at the therapeutic goal of anticoagulation). (3) Malignant tumors. (4) Patients with neurological system diseases that have an obvious impact on this trial, such as Parkinson’s disease, convulsive epilepsy, traumatic brain injury (TBI), or severe neuromuscular disorders. (5) Patients with rheumatoid arthritis, fractures, or other trauma that may confound the efficacy assessment. (6) Patients have received anti-spasticity medications within the past 1 month, such as baclofen, dantrolene, tizanidine, and botulinum toxin. (7) Patients who are allergic to acupuncture or have a history of severe allergic predisposition. (8) Female patients who are pregnant, breastfeeding, or planning pregnancy during the trial period. (9) Patients enrolled in other acupuncture or pharmacotherapy trials.

### 2.3 Recruitment process

Participants will be recruited through both online and offline channels. Recruitment notices will be posted on the hospital’s official website, and interested individuals may contact the research team via WeChat or telephone. Concurrently, trained research assistants will screen potential participants directly in hospital wards and outpatient clinics.

### 2.4 Patient adherence

To ensure the completion of the trial, measures must be implemented to enhance patient compliance. First, researchers will comprehensively review the pathophysiology, diagnostic criteria, and current treatment strategies for PSS prior to trial initiation and ensure comprehensive empathetic communication with participants to establish mutual trust. Second, we will inform patients that acupuncture treatment is conducted on the basis of conventional treatment and rehabilitation training and it does not endanger their health to mitigate the participants’ concerns. Third, all acupuncturists involved in the trial will receive professional and standardized training before enrollment to ensure proficiency. Furthermore, all acupuncture treatments will be performed by a designated certified acupuncturist to guarantee that the patients have a favorable experience. Additionally, our research team will maintain regular, transparent communication with participants and their caregivers to foster a shared understanding of the patient’s clinical condition and encourage them to receive active engagement in the acupuncture treatment. Patients will be clearly informed that all trial-related examinations (such as surface electromyography (sEMG) and shear-wave ultrasound elastography), scale assessments, and 12 acupuncture treatments are free of charge. Finally, all adverse events and participant dropouts during the trial will be documented.

### 2.5 Randomization, allocation concealment, and blinding

Eligible trial participants will be randomly assigned to one of three groups—acupuncture, sham acupuncture, or conventional treatment—using block randomization implemented in the Statistical Package for Social Sciences (SPSS, IBM Corp., NY, USA), version 27. Stratification will be performed according to sex, age, disease duration, and stroke subtype. Randomization codes will be generated by an independent research assistant, placed in sequentially numbered, opaque, sealed envelopes, and distributed to participants only after the completion of baseline assessments.

Participants, outcome assessors, and statisticians will remain blinded to the group allocation throughout the trial. Given the nature of acupuncture intervention, acupuncturists could not be blinded. Blinding codes were securely maintained by an independent researcher who was not involved in participant recruitment, treatment delivery, or outcome assessment. To preserve blinding integrity, participants will be assessed and treated in individual quiet rooms equipped with partition curtains to prevent communication among participants.

### 2.6 Intervention

**Figure 2:**
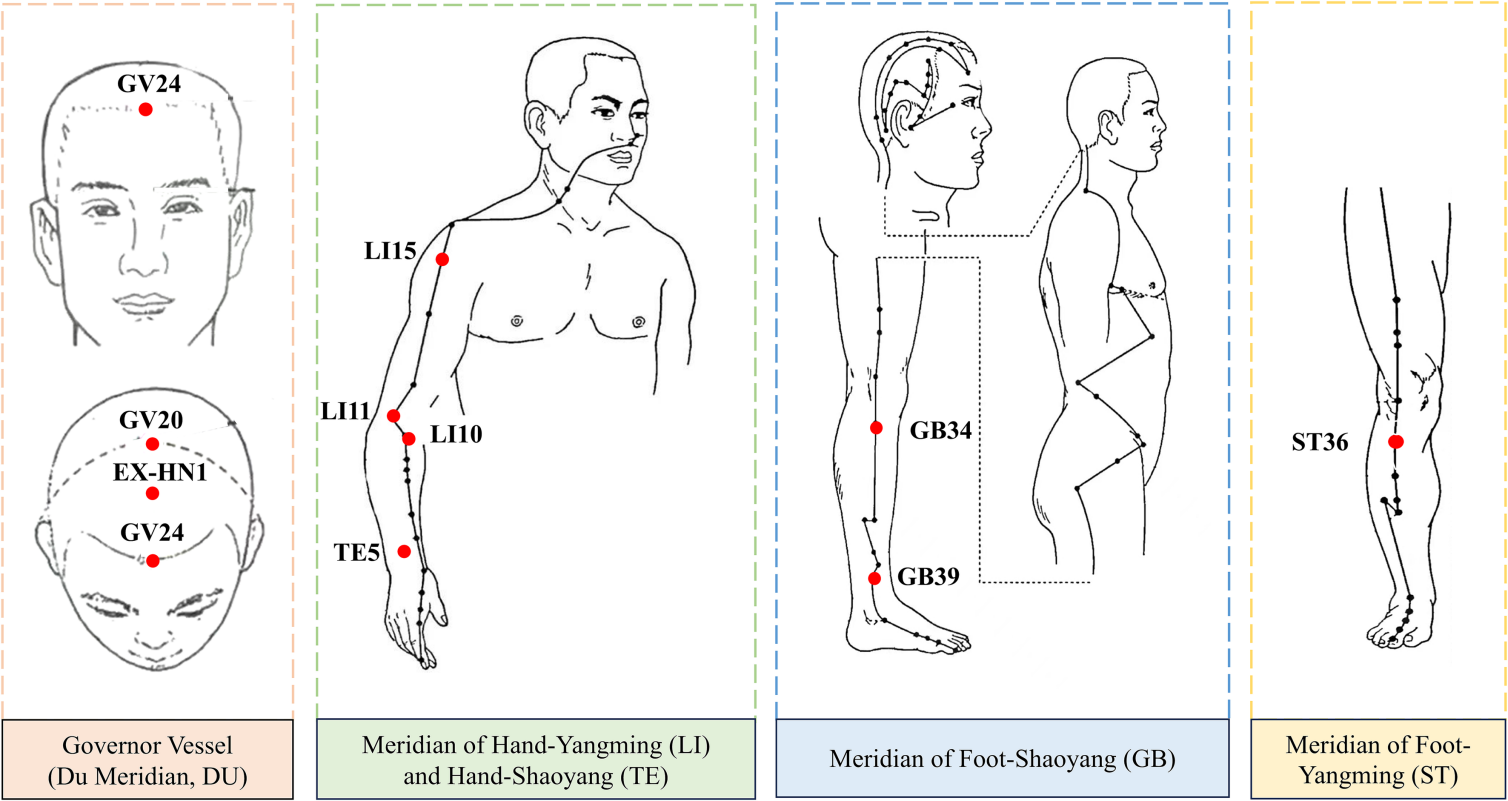
Locations of acupoints.

Patients in these three groups are required to receive conventional therapy and rehabilitation training recommended by the Stroke Prevention and Treatment Guideline 2024 (SPTG 2024) (17). Acupuncture treatment will be performed by acupuncturists who hold a Chinese medicine practice license issued by the National Health Commission of the People’s Republic of China and have 5 years of acupuncture clinical experience. Locations of acupoints are displayed in Table 2 and Figure 2.

**Table 2:** Acupuncture point locations.

Acupoints	Location
GV20 (Baihui)	On the head, 5 cun directly above the midpoint of the anterior hairline at the midpoint of the line connecting the apexes of both ears.
GV24 (Shenting)	On the head, 0.5 cun directly above the midpoint of the anterior hairline.
EX-HN1 (Sishencong)	On the vertex of the head, 1 cun anterior, posterior, and lateral to Baihui (GV20).
LI10 (Shousanli)	On the radial side of the dorsal surface of the forearm and on the line connecting Yangxi (LI5) and Quchi (LI11), 2 cun below the cubital crease.
LI11 (Quchi)	On the lateral aspect of the elbow, at the midpoint of the line connecting LU5 with the lateral epicondyle of the humerus.
LI15 (Jianyu)	On the shoulder, superior to the deltoid muscle, in the depression anterior and inferior to the acromion when the arm is abducted or raised to the shoulder level.
TE5 (Waiguan)	On the posterior aspect of the forearm, 2 cun proximal to the dorsal wrist crease at the midpoint between the ulna and radius.
ST36 (Zusanli)	3 cun directly below Dubi (ST35) in the depression, one fingerbreadth lateral to the anterior crest of the tibia.
GB34 (Yanglingquan)	In the depression anterior and inferior to the head of the fibula at the tibiofibular articulation.
GB39 (Xuanzhong)	On the lateral side of the leg, 3 cun above the tip of the external malleolus on the anterior border of the fibula.

#### 2.6.1 Acupuncture group

The treatment plan will be developed based on previous literature (18) and consensus among acupuncture experts. The following acupoints will be selected: Baihui (GV20), Shenting (GV24), Sishencong (EX-HN1), Jianyu (LI15), Quchi (LI11), Shousanli (LI10), Waiguan (TE5), Yanglingquan (GB34), Zusanli (ST36), and Xuanzhong (GB39). The head and impaired limb will be exposed, and the skin at the acupoints will be disinfected with 75% alcohol, and 3 M adhesive plastic pads will be applied to the acupoints. These acupoints will be punctured with disposable stainless steel needles (0.25 mm × 40 mm, Andi) into the subcutaneous tissue or the galea aponeurotica of the head through these pads. The procedure will be as follows: Baihui (GV20), Shenting (GV24), and Sishencong (EX-HN1) will be punctured to a depth of 0.5–1 cun (a cun is a traditional Chinese proportional body-based unit used in acupuncture) at an angle of 15–30°, and the needles will then be manipulated by twisting for 2 min at a frequency of 200 twists/min. For Jianyu (LI15), Quchi (LI11), and Yanglingquan (GB34)—located around the shoulder, elbow, and knee joints, respectively—the needles will be punctured perpendicularly at a depth of 1–1.5 cun to achieve deqi (sensation of soreness, distension, and heaviness); then the needles will be withdrawn to the subcutaneous layer. Subsequently, the needles will be redirected and punctured obliquely to a depth of 1 cun, and the acupuncturist will assist patients in performing slow movements of the shoulder, elbow, and knee joints to gradually relieve spasticity. The other acupoints will be punctured perpendicularly to a depth of 1 cun and manipulated using lifting, thrusting, and rotating to facilitate deqi. Each treatment session will last 25–30 min, and during the needle retention period, the needles will be manipulated by lifting, thrusting, twirling, and rotating every 10 min to maintain the deqi sensation. Each participant will receive 12 sessions of acupuncture treatment (three times per week).

#### 2.6.2 Sham acupuncture group

Participants assigned to this group will receive non-penetrating sham acupuncture at the same acupoints as the acupuncture group. The blunt-tip retractable needle (0.25 mm × 40 mm) was fixed on the acupoint through a 3 M adhesive plastic pad. When the acupuncturist pressed the needle, the blunt tip contacted the skin without penetrating it, producing only a pricking sensation. Simultaneously, the needle body retracted into the handle, creating the “visual mimicry” of penetration. Participants in this group will undergo the same needle manipulation, treatment frequency, and duration as those in the acupuncture group. The needles used in the sham group were identical in appearance, material, and size to the real ones, and the 3 M plastic pads were matched in shape, color, and application method.

#### 2.6.3 Basic treatment group

Participants will not receive acupuncture treatment in this group but will only receive conventional therapy and rehabilitation training recommended by the Stroke Prevention and Treatment Guideline 2024 (SPTG 2024). Patients in this group who complete follow-up are eligible for a complimentary 4-week (12 sessions) acupuncture treatment.

### 2.7 Outcome measurements

#### 2.7.1 Primary outcome

In this trial, the primary outcome will be defined as the change in Modified Ashworth Scale (MAS) scores from baseline to week 4 of treatment. The MAS is a validated clinical tool used to assess the severity of muscle spasticity and evaluate treatment efficacy (19, 20). It comprises six ordinal grades—0, I, I+, II, III, and IV—which correspond to numerical scores of 0, 1, 2, 3, 4, and 5, respectively. Higher grades and scores indicate greater spasticity severity. In this study, MAS assessments will be conducted bilaterally on the biceps brachii and tibialis anterior muscles of patients with PSS.

#### 2.7.2 Secondary outcomes

##### 2.7.2.1 Fugl-Meyer assessment

The Fugl-Meyer Assessment (FMA) is a standardized and validated tool widely used to comprehensively evaluate motor impairment following neurological injury (21). The FMA includes five dimensions (motor function, sensation, balance, range of motion, and joint pain). The upper limb score ranges from 0 to 66, with the lower limb score ranging from 0 to 34; the more severe the symptoms, the lower the score.

##### 2.7.2.2 Clinical spasticity index

The Clinical Spasticity Index (CSI) is used for the quantitative assessment of muscle spasticity. CSI includes tendon reflexes, muscle tone, and clonus, with a score ranging from 0 to 16, in which 0–9 indicates mild spasticity, 10–12 indicates moderate spasticity, and 13–16 indicates severe spasticity.

##### 2.7.2.3 National Institutes of Health Stroke Scale

The National Institutes of Health Stroke Scale (NIHSS) is used to assess the severity of neurological impairment in patients with stroke, to evaluate prognosis, and to monitor disease progression. It consists of 11 items with total scores ranging from 0 to 42, in which 0–1 means normal or nearly normal, 1–4 means minor stroke, 5–15 means moderate stroke, 15–20 means moderate to severe stroke, and 21–42 means severe stroke.

##### 2.7.2.4 Modified Barthel Index

The Modified Barthel Index (MBI) is commonly used to assess activities of daily living (22). It has 10 items covering feeding, bathing, dressing, grooming, transferring, and other factors, with a total score of 100. Higher MBI scores indicate a higher quality of life.

##### 2.7.2.5 Surface electromyography

Surface electromyography (sEMG) is an important indicator to evaluate neuromuscular activity under static and dynamic states, with the advantages of being non-invasive, real-time, and capable of multi-target measurement (23). Studies have indicated that changes in sEMG parameters correlate with limb dysfunction (assessed by FMA) and spasticity (assessed by MAS) (24). Integrated electromyography (IEMG), median frequency (MF), and root mean square (RMS) will be used as indicators for efficacy evaluation in this study.

##### 2.7.2.6 Shear-wave ultrasound elastography

Shear-wave ultrasound elastography (SWUE) is used to quantitatively assess the mechanical properties of muscles, such as stiffness, elasticity, and changes in echogenicity. It is helpful to judge the definitive diagnosis and effectiveness of treatment (25, 26). The maximum modulus values (Emax) will be measured and evaluated before and after the treatment in this trial.

In this study, the FMA, CSI, NIHSS, and MBI scores will be assessed at baseline, 2 weeks, 4 weeks, and during follow-up. MAS, sEMG, and SWUE will be evaluated at baseline and 4 weeks. The integration of MAS and CSI with sEMG and SWUE enables a comprehensive assessment of spasticity severity, muscle electrophysiological activity, and morphological characteristics (e.g., muscle fiber thickness and echo intensity). Meanwhile, the FMA, NIHSS, and MBI collectively focused on evaluating improvements in neurological function and the enhancement of real-world quality of life.

### 2.8 Safety and adverse events

The acupoints selected in this trial are based on a consensus among acupuncture experts and are considered relatively safe. Potential adverse events included pain, numbness, subcutaneous swelling, bruising, fainting during acupuncture, needle sticking, or needle bending. If an adverse event occurs, it will be recorded in detail in the Case Report Forms (CRFs). Serious adverse events, such as syncope, deep tissue infection, or abscess, will be promptly reported to the Ethics Committee, and affected participants will be immediately withdrawn from the trial and provided with active and effective treatment.

### 2.9 Blind success rate evaluation

A blinded assessment will be conducted 5–10 min after the final treatment session in the 4 weeks for both the acupuncture and sham acupuncture groups. Patients will complete a questionnaire indicating whether they believed they received acupuncture, with response options of “Yes,” “No,” or “Uncertain.” The number of responses in each category will be recorded separately. Then, the Bang’s Blinding Index (BBI) will be used to calculate for each group according to Bang’s index formula (27). BBI values ranged from −1 to 1, with results closer to −1 indicating more successful blinding. A BBI value of < 0.2 was considered indicative of successful blinding. In addition, the recruitment rate and compliance of the patients will be statistically analyzed.

### 2.10 Quality control and data management

Outcome assessors will collect all participant data using paper-based CRFs and subsequently enter the data into an electronic database. The research team will conduct periodic data monitoring to verify completeness, accuracy, and authenticity while concurrently tracking trial progress and adherence to the intervention protocol. To enhance adherence, researchers will maintain regular contact with the participants via telephone or WeChat, providing reminders to actively engage in treatment, scheduled assessments, and follow-up visits. All adverse events and reasons for participant discontinuation—whether from the trial or from follow-up—will be systematically documented.

Acupuncturists, outcome assessors, and statisticians will all receive standardized training on the study protocol to ensure a full understanding of the study procedures and details. Specifically, acupuncturists will complete comprehensive professional training covering acupoint selection and localization, acupuncture techniques, treatment frequency and duration, and distinct protocols for both verum and sham acupuncture, thereby ensuring intervention consistency and fidelity across all participants. All members of the research team will attend scheduled organizational meetings every three weeks to review the progress, identify challenges, and collaboratively resolve issues arising during the trial.

### 2.11 Sample size calculation

This trial is an exploratory study. Based on the relevant literature (28, 29) and our preliminary pilot trial, it was predicted that the mean reduction in MAS scores for the acupuncture, sham acupuncture, and basic treatment groups at the end of the intervention would be 2.58, 1.00, and 2.19, with standard deviations of 1.36, 1.62, and 1.48, respectively. The trial employs a 1:1:1 parallel design, with *α* = 0.05 and 1−*β* = 0.9. Considerations for the feasibility of performing this trial and the calculated sample size required per group are 48 cases using the PASS 15.0 software. Accounting for a potential dropout rate of 20%, each group will require 60 participants, resulting in a total sample size of 180 cases.

### 2.12 Statistical analyses

All statistical analyses will be performed using IBM SPSS Statistics 25.0. Intention-to-treat (ITT) analysis will be applied to all randomized participants, with missing data handled through multiple imputation methods. Normality tests will be conducted on all data, and continuous variables with a normal distribution will be presented as the mean±SD. Otherwise, the data will be expressed as M (P25-P75). For comparisons among the three groups, continuous variables will be analyzed using parametric tests (ANOVA) or non-parametric tests (Kruskal-Wallis) based on the data features. Categorical variables will be compared using the chi-square test. A *p* value of <0.05 will be defined as statistically significant. Efficacy analyses will be performed on both intention-to-treat (ITT) and per-protocol (PP) populations, with the ITT set including all randomized participants analyzed according to their allocated groups, regardless of protocol deviations, whereas the PP set will consist of participants who comply with the trial procedures without major deviations.

## 3 Discussion

Recent studies have suggested that acupuncture may exert beneficial effects on PSS (13, 14). However, the reliability of these findings remains uncertain, and they fail to yield robust conclusions owing to methodological limitations, including small sample sizes, suboptimal randomization procedures, and inadequate blinding. To date, large-scale, rigorously designed randomized controlled trials evaluating the efficacy and safety of acupuncture for PSS are lacking. This study aims to generate high-quality evidence regarding the therapeutic potential of acupuncture in patients with PSS.

### 3.1 Design of trial intervention

Acupoint selection for this trial is based on expert consensus from the multidisciplinary fields of acupuncture, rehabilitation, and evidence-based practice. According to acupuncture theory, acupoints have local therapeutic effects, meaning that each acupoint is able to treat diseases in that area and adjacent tissues and organs. Since the brain is the primary lesion site in PSS, Baihui (GV20), Shenting (GV24), and Qianshencong (Sishencong, EX-HN1) are selected as core acupoints. These acupoints are located on the head and are intended to ameliorate cerebral neural damage (30) and enhance the efficacy of acupuncture treatment. Furthermore, the combination of acupoints is capable of enhancing their functional correlations and generating superior effectiveness, which is a crucial component in alleviating spasticity and improving efficacy (31). In addition to the above acupoints, Jianyu (LI15), Quchi (LI11), Zusanli (ST36), Yanglingquan (GB34), and Xuanzhong (GB39) were selected. Previous studies indicate that these acupoints not only harmonize qi and blood circulation in the meridians and nourish the tendons, but also modulate peripheral sensory input to the central nervous system, thereby enhancing cortical excitability and promoting neuroplasticity in motor-related regions (32). To control for potential acupuncture-related confounding factors during the trial, minimize sources of bias and measurement imprecision that could influence clinical outcomes, and enhance the authenticity and reliability of the findings, blunt-tipped telescopic needles are employed to ensure participant blinding. In addition, we set up a basic treatment group (receiving only conventional therapy and rehabilitation training) as a reference to the acupuncture and sham acupuncture groups, thereby improving the clinical applicability of the study results.

### 3.2 Multidimensional efficacy evaluation model

Currently, the assessment of clinical outcomes for PSS primarily relies on scales such as MAS, FMA, and CSI. The sensitivity and accuracy of scale assessments have improved compared to those of the past (33). The evaluation results still heavily depend on the assessor’s experience and understanding of the scale’s grading criteria (34), making it impossible to eliminate the influence of subjective factors. As tools reflecting effect mechanisms, sEMG and SWUE have been widely used to assess limb dysfunction after stroke, with recorded muscular activity information (such as nerve conduction velocity, amplitude, and electrical activity) during movement and rest states, and to perform analyses to evaluate dynamic muscle deformation. This study analyses spastic muscles quantitatively by collecting IEMG, MF, RMS, and SWUE data, which serve as important indicators for evaluating muscle structural changes and the effects of acupuncture intervention. Combined with clinical scales, a multidimensional evaluation of the efficacy of acupuncture for PSS is conducted from both subjective and objective perspectives. It aims to help overcome the limitations of a singular efficacy evaluation and inadequate neuromuscular mechanism explanation, facilitating dynamic and visual analysis of the mechanisms of the acupuncture effect.

### 3.3 Limitations

This study has several limitations. First, as a single-center, exploratory, randomized controlled trial conducted primarily at one hospital in Beijing, it may not fully capture the diversity of the patient population, which may lead to selection bias. Thus, large-scale, multi-center RCTs are needed to confirm the definite efficacy of acupuncture for PSS in the future. Second, the age of participants was restricted to 35–75 years to ensure relative pathophysiological homogeneity within the study population and to minimize age-related confounding. Although this design strengthens internal validity, it limits the direct extrapolation of the findings to older patient populations. Third, given the restrictions of equipment, cost, and time, the study mainly emphasized the neuromuscular effect mechanisms of acupuncture without delving into the central regulatory mechanisms or exploring acupuncture-induced neural plasticity. Finally, given the complex nature of acupuncture intervention, blinding of the acupuncturists was not feasible, which may have introduced performance bias and affected the interpretation of the results.

## 4 Conclusion

In summary, this study will evaluate the clinical efficacy and safety of acupuncture in treating post-stroke spasticity and explore the neuromuscular mechanisms by which acupuncture ameliorates spasticity.
